# A randomized cross-over trial on the direct effects of oxygen supplementation therapy using different devices on cycle endurance in hypoxemic patients with Interstitial Lung Disease

**DOI:** 10.1371/journal.pone.0209069

**Published:** 2018-12-28

**Authors:** Anne Edvardsen, Inga Jarosch, Anita Grongstad, Laura Wiegand, Rainer Gloeckl, Klaus Kenn, Martijn A. Spruit

**Affiliations:** 1 Department of Respiratory Physiology, LHL-hospital Gardermoen, Jessheim, Norway; 2 Department of Respiratory Medicine & Pulmonary Rehabilitation, Schoen Klinik Berchtesgadener Land, Schoenau am Koenigssee, Germany; 3 Department of Internal Medicine, Philipps University of Marburg, Marburg, Germany; 4 Department of Prevention, Rehabilitation and Sports Medicine, Technical University of Munich (TUM), Munich, Germany; 5 Department of Pulmonary Rehabilitation, Philipps University of Marburg, Center for Lung Research (DZL), Marburg, Germany; 6 Department of Research and Education, CIRO+, Centre of Expertise for Chronic Organ Failure, Horn, The Netherlands; 7 REVAL—Rehabilitation Research Center, BIOMED—Biomedical Research Institute, Faculty of Medicine and Life Sciences, Hasselt University, Diepenbeek, Belgium; 8 Department of Respiratory Medicine, Maastricht University Medical Centre, NUTRIM School of Nutrition and Translational Research in Metabolism, Maastricht, The Netherlands; Edge Hill University, UNITED KINGDOM

## Abstract

**Background:**

In patients with interstitial lung disease (ILD) a cardinal feature is exercise intolerance, often associated with significant dyspnea and severe hypoxemia. Supplemental oxygen therapy may be offered during exercise. The Oxymizer is a nasal cannula with an incorporated reservoir with the potential to deliver higher oxygen doses to the patient.

**Objective:**

The primary aim was to investigate the effect of supplemental oxygen delivered via Oxymizer compared to a conventional nasal cannula (CNC) in patients with ILD during constant work rate tests (CWRT). Secondary aim was to evaluate effects on oxygen saturation (SpO_2_), dyspnea and heart rate at isotime.

**Methods:**

In this randomized crossover study 24 ILD patients established on long-term oxygen treatment were included. Patients performed four cycling CWRT at 70% of their peak work rate; twice with the Oxymizer and twice with the CNC.

**Results:**

Twenty-one patients finished all CWRTs (age 60 ± 10.9 years, VC 55.4 ± 23.0%predicted). Cycle endurance time was significantly higher while using the Oxymizer compared to CNC (718 ± 485 vs. 680 ± 579 seconds, p = 0.02), and SpO_2_ at isotime was significantly higher while using the Oxymizer (85.5 ± 6.7 vs. 82.8± 7.2, p = 0.01). Fifteen of the 21 (71%) patients cycled longer with the Oxymizer. There were no significant differences for dyspnea and heart rate.

**Conclusions:**

Supplemental oxygen provided by the Oxymizer significantly, but modestly, improved cycle endurance time and SpO_2_ at isotime in ILD patients compared to CNC.

## Background

Dyspnea is a cardinal feature of patients with ILD, which is usually associated with significant exercise intolerance [1–3]. Moreover, as a result of an impaired gas exchange at the lungs, severe hypoxemia is frequently seen both at submaximal and maximal exercise [4,5]; and oxygen desaturation during walking is associated with increased mortality in patients with ILD [6]. Therefore, supplemental oxygen therapy may be offered during exercise in ILD patients with exercise-induced desaturation to reduce dyspnea and extend the exercise duration [7]. Indeed, a correction of exercise-induced oxygen desaturation may play an important role for ILD-patients to become and sustain physically active [8].

Thus far, there is a lack of data to guide the best management of exercise-induced symptoms [2,3]. According to the British Thoracic Society oxygen guidelines, the treatment goal for oxygen supplementation therapy is to achieve arterial oxygen saturation (SpO_2_) >90% [7]. However, this may be challenging since ILD patients often require high oxygen flow rates. There are only a few prospective studies on the effects of supplemental oxygen therapy during exercise in patients with ILD [9,10], which improves maximum oxygen uptake, maximal exercise workload and exercise duration compared to room air [2, 9–11]. However, a recent Cochrane review was inconclusive due to a limited number of studies (n = 3) [12]. Supplemental oxygen therapy during exercise is usually provided by a conventional nasal cannula (CNC) from either a continuous flow or from an oxygen-conserving device. An alternative is to administer oxygen using an oxygen-conserving reservoir. The Oxymizer was first described in the 1980s [13], and is an oxygen-conserving reservoir designed to accumulate the continuous flow of oxygen normally wasted during exhalation. The saved oxygen is available as a bonus at the very beginning of each inhalation cycle and the device concentrates oxygen delivery when the delivered oxygen can participate in alveolar-capillary gas exchange [14]. This may lower flow rates and thereby reduce oxygen costs and even more important increase ambulation by making portable system last longer [15–17]. Moreover, supplemental oxygen therapy administered by a Oxymizer improves resting and exercise arterial oxygen tension and saturation when compared to CNC in selected patients with chronic obstructive pulmonary disease (COPD) [16–18] and for a small number of patients with ILD [19]. However, the effect of breathing supplemental oxygen therapy with Oxymizer compared to CNC during exercise has not been studied in patients with ILD. Nevertheless, it seems reasonable to hypothesize that the average cycle endurance time would be significantly higher when using the Oxymizer compared to CNC.

Therefore, the primary aim was to compare the effects of supplemental oxygen therapy given by CNC *versus* an Oxymizer on cycle endurance time in patients with ILD. Our secondary aim was to assess effects of the two different nasal cannulas on SpO_2_, partial pressure of carbon dioxide (PCO_2_), and heart rate (HR) at isotime during a constant work rate test.

## Methods and materials

### Patients

All patients with ILD according to international consensus classification of idiopathic interstitial pneumonias [20] who were referred for a 3-week, comprehensive, inpatient pulmonary rehabilitation program [21] and were established on long-term oxygen therapy (LTOT) with oxygen flow rates ≥ 2 liter per minute (l/min) at rest or during activity, were eligible for participation in the study. Baseline oxygen prescription were set by the corresponding physician. All patients used their regular medication and were in a stable phase of the disease. Absolute and relative contraindications for cardiopulmonary exercise testing [22] and musculoskeletal disease were exclusion criteria.

### Study design

**Fig 1:**
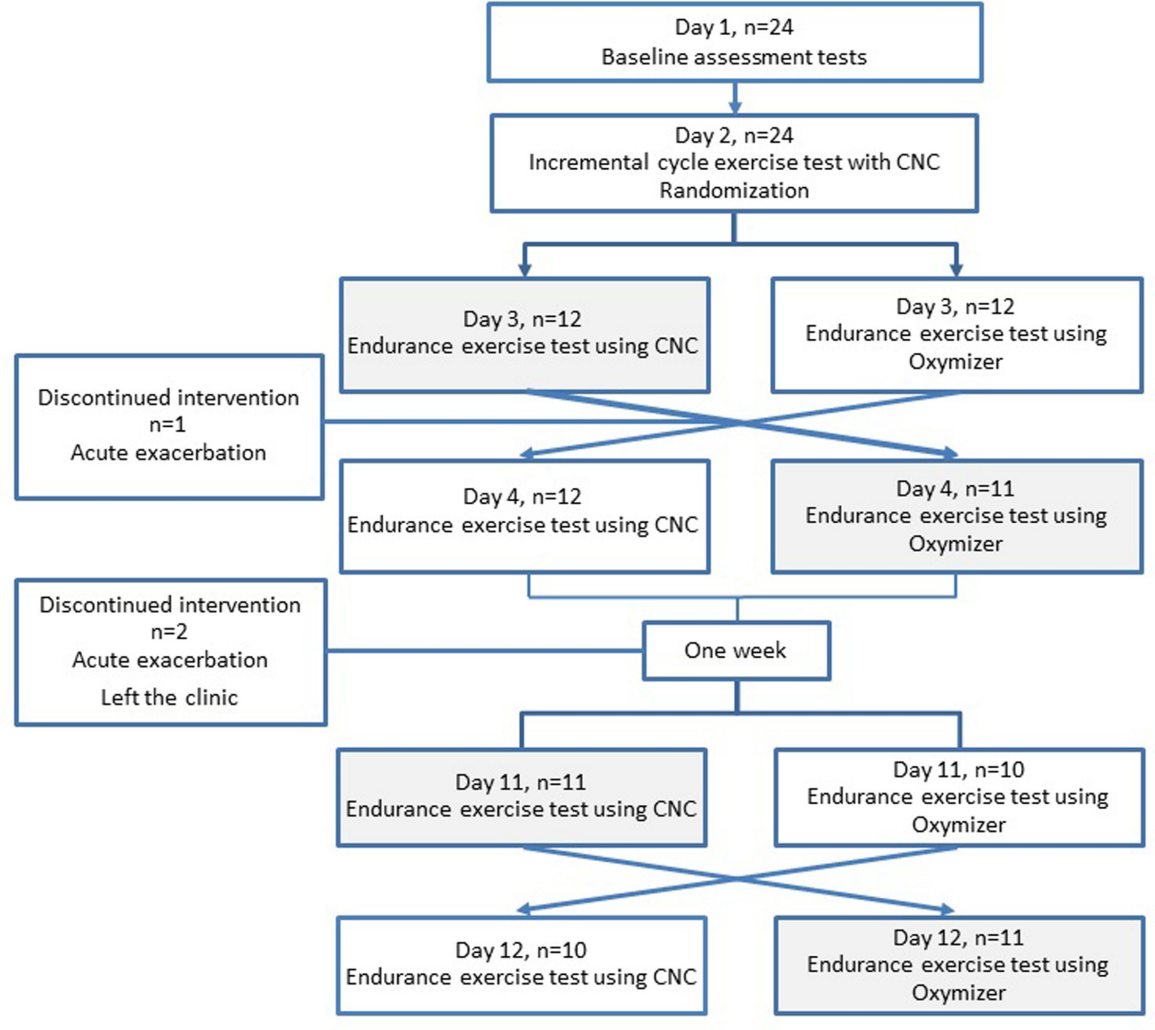
Flow-chart of the intervention.

**Fig 2:**
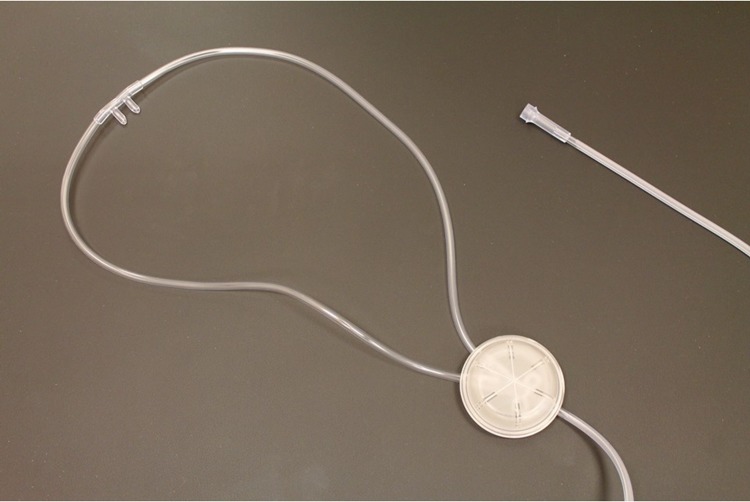
The Oxymizer pendant used during the constant work rate tests (CWRT).

In this randomized cross-over trial, 24 patients with ILD were from February to December 2012 consecutively included after given their written informed consent (Fig 1). The main study procedure consisted of a constant work rate test (CWRT) on a bicycle with supplemental oxygen administered in random order from either a CNC or Oxymizer pendant (Fig 2). All patients used their individualized oxygen flow rate during exercise ranging from 2 to 6 l/min with the CNC. The same dose was given during the CWRTs with CNC and Oxymizer, respectively. The FiO_2_ from Oxymizer is estimated to be approximately the twice as increase in FiO_2_ with the CNC. For example 2 L/min on CNC will give approximately 27% FiO_2_ and 33% FiO_2_ with the Oxymizer.

At baseline, pulmonary function testing (Master Screen Body Plethysmograph, Jaeger, Germany), capillary blood gas analyses (ABL 820, Radiometer, Denmark) and body composition (NutriGuard MS, Data Input Body Composition, Germany) were performed. The study was approved by the Bavarian Ethics Committee (ID12019) and listed in the Clinical Trials Registry (www.clinicaltrials.gov, NCT01713413).

### Outcomes and measurements

The primary outcome was endurance time at the CWRT (seconds). Secondary outcomes were SpO_2_, PaCO_2_ and HR at isotime of the CWRT. Isotime was defined as the duration of the shortest CWRT between the tests with the CNC and the Oxymizer. In addition, SpO_2_, HR, partial pressure of O_2_ (PaO_2_), PaCO_2_, dyspnea and leg fatigue were assessed at end-exercise.

### Functional tests

After baseline assessment on day 1, all subjects performed day 2 a standardized symptom-limited incremental test on a cycle ergometer (Cardiomed Bike, Proxomed Medical, Germany), starting at 25 Watt and increased with 10 Watt every minute until exhaustion. Subjects were highly encouraged to reach their peak work rate (PWR); defined as the highest work rate the subjects were able to maintain for 60 seconds. On day 3 and 4, the subjects performed a CWRT at 70% of their PWR using CNC or Oxymizer in random order. One week later, to compensate for potential day to day variances, they repeated the two CWRTs on two consecutive days at the same workload using CNC and Oxymizer in reverse order. For all analysis were mean of the outcomes from the CWRTs with the two devices used. Subjects were instructed to cycle as long as possible maintaining ≥ 50 revolutions per minute. Encouraging comments were given every minute. If the subjects were able to cycle more than 30 minutes, intensity was increased by 10 Watt every 5 minute until exhaustion. The subjects were told to inhale through the nose. Settings of the cycle ergometer (e.g. seat height) were the same for all tests. HR, SpO_2_ and transcutaneous pCO_2_ were continuously measured by SenTec Digital Monitoring System via an ear clip (Sentec AG, Switzerland). Dyspnea and leg fatigue were measured by Borg CR10 scale [23] at rest sitting on the cycle ergometer and at the end of the CWRT. Capillary samples for blood gases were assessed from a hyperemic earlobe and immediately analyzed (ABL 820, Radiometer, Denmark). Randomization was performed by computer-generation. Due to the study design with two different looking devices it was not possible to blind neither patients nor the investigators who performed the CWRTs. However, the researchers who performed the analysis were blinded to group allocation.

### Statistical analyses

A sample size calculation was performed (t-test, standard deviation 125 s); when using a statistical power of 80% and a risk for a type I error (α) < 5%, a sample size of 24 patients was calculated to detect a minimal important difference of 105 s [24] for the CWRT between the two O_2_-supplementation devices.

Data are presented as mean and standard deviation (SD). A p-value <5% was considered as statistical significant. Continuous and outcome variables were checked for normality prior to analyses. Mean values from the two CWRTs with CNC and Oxymizer, respectively, were used in the final analyses. Paired t-tests were used for intra-group comparisons. Data analysis was performed using IBM SPSS Statistics v.22.

## Results

Twenty-four patients (38% women) with ILD were included. All patients performed the first two CWRTs, and 21 patients performed all four CWRTs (Fig 1). Baseline characteristics did not change significantly including the three excluded patients (Table 1). These patients were excluded from all analyses.

**Table 1:** Patient baseline characteristics, n = 21.

Variables	
Sex, Male/Female	13/8 (62%/38%)
Age, yrs	60.1 ± 10.9
BMI, kg/m^2^	26.9 ± 4.6
FFMI	18.9 ± 2.7
**Diagnosis**	
IPF	9 (43%)
EAA	3 (14%)
NSIP	3 (14%)
RA	2 (10%)
Other fibrosis diagnosis	4 (19%)
**Lung function**	
VCin, L	2.0 ± 0.9
VCin, %pred	55.4 ± 23.0
FEV_1_, %pred	57.4 ± 19.1
FEV_1_/FVC	87.6 ± 9.7
*D*L,CO, mmol•min^-1^•kPa^-1^	1.8 ± 1.2
*D*L,CO, %pred	20.1 ± 12.4
TLC, L	4.4 ± 1.4
TLC, %pred	73.1 ± 22.1
**Baseline blood gases and pulse oximetry**	
*Room air*	
PaO_2_, kPa	7.3 ± 1.0
PaCO_2_, kPa	5.4 ± 0.8
*With supplemental oxygen*	
O_2_-flow, L/min	4.0 ± 1.3
PaO_2_, kPa	10.6 ± 2.6
PaCO_2_, kPa	5.5 ± 0.9
SpO_2_, %	97.4 ± 3.2

Data are normally distributed and presented as n (%) and mean (SD). BMI: body mass index; IPF: Interstitial pulmonary fibrosis; EAA: Exogen allergic alveolitis; NSIP: Non-specific interstitial pneumonia; RA. Rheumatoid arthritis; FEV_1_%pred: forced expiratory volume in one second in per cent of predicted; FFMI: Fat-free mass index; FVC: forced vital capacity; *D*L,CO: diffusing capacity of the lung for carbon monoxide; TLC: total lung capacity; PaO_2_: arterial oxygen pressure; PaCO_2_: arterial carbon dioxide pressure; SpO_2_: arterial oxygen saturation by pulse oximetry; VCin: inspiratory vital capacity.

On average, the subjects had severely reduced vital capacity, a reduced total lung capacity, a reduced diffusion capacity, mild hypoxemia when breathing room air, and were normocapnic.

The peak work rate obtained during the initial incremental cycling test was 74 ± 21 Watts. The mean oxygen supplement dose given with both CNC and Oxymizer was 4.0 ± 1.4 l/min.

**Fig 3:**
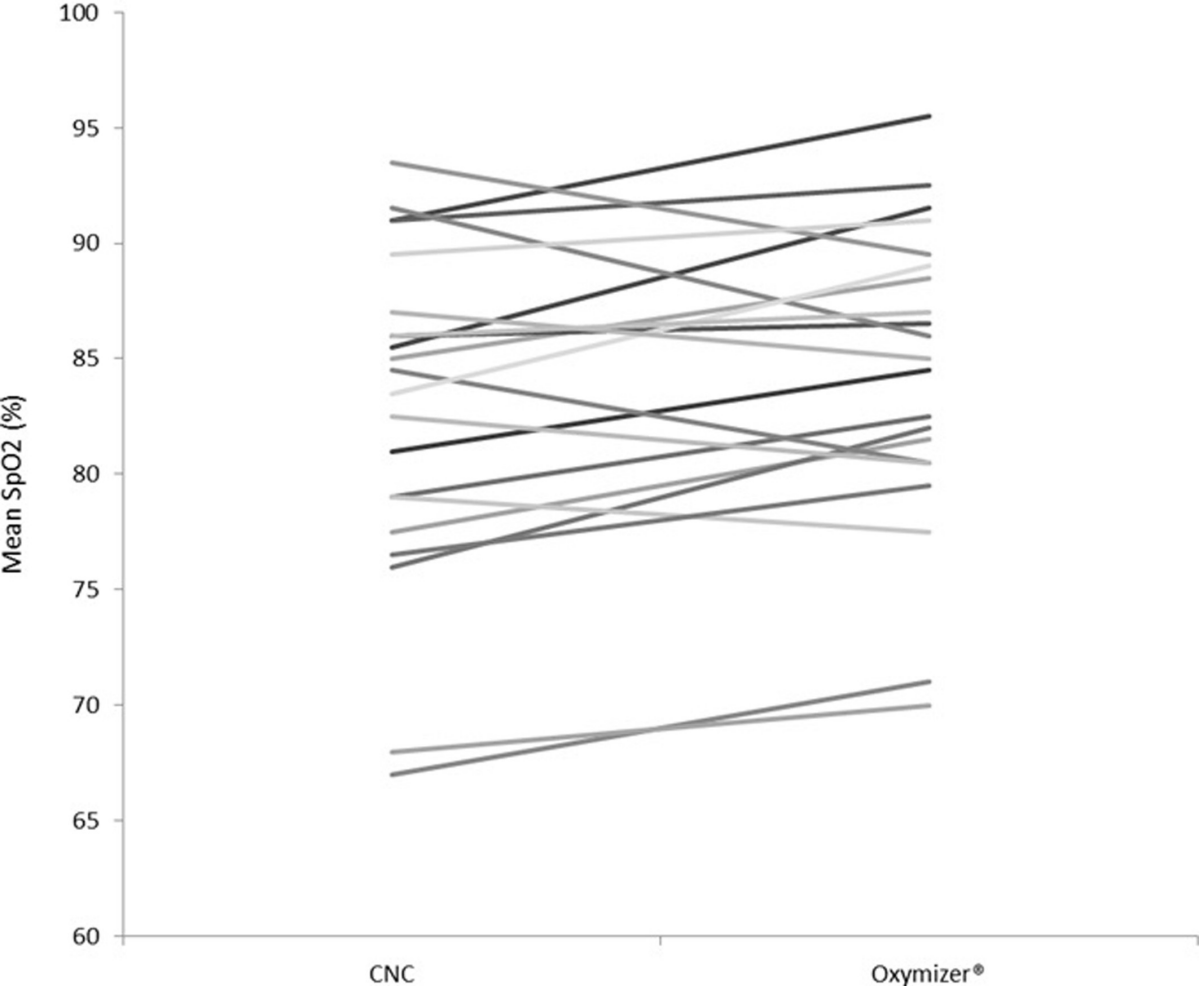
Individual mean SpO_2_ at isotime for the two CWRT with CNC and Oxymizer respectively. CWRT: Constant work rate test; CNC: Conventional nasal cannula, n = 21.

Patients were able to cycle significantly longer while using the Oxymizer than with CNC (mean difference between devices: 38 ± 265 seconds; p = 0.02 (Table 2)). 15 of the 21 (71%) patients increased cycle endurance time, one cycled equal time and five cycled for a shorter duration while using the Oxymizer compared to the CNC. All patients except one, showed a pre-exercise SpO_2_ higher than 94% with supplemental oxygen therapy on both devices. SpO_2_ was significantly higher for the Oxymizer at isotime (85.5 ± 6.7% vs. 82.8 ± 7.2, p = 0.01), but no significant difference was seen for SpO_2_ at end-exercise between the two oxygen devices (Table 2 and Fig 3). Neither CNC nor Oxymizer managed to sufficiently oxygenate the patients according to the guidelines. SpO_2_ dropped below 90% at end-exercise for 81% of the patients for both the CNC and Oxymizer.

**Table 2:** Constant work rate test (CWRT) data, n = 21.

Variables	CNC	Oxymizer	p-value
*Pre-exercise*			
SpO_2_, %	97.5 ± 2.7	97.9 ± 1.9	0.38
PaO_2_, kPa	12.4 ± 3.4	13.0 ± 3.9	0.35
PaCO_2_, kPa	5.2 ± 0.8	5.4 ± 0.9	0.10
HR, bpm	89 ± 19	88 ± 23	0.59
Dyspnea, Borg	2.3 ± 1.8	2.3 ± 1.8	0.19
Leg fatigue, Borg	1.8 ± 1.6	1.6 ± 1.4	0.67
*End-exercise*			
Endurance time, sec	680 ± 579	718 ± 485	0.02
SpO_2_, %	82.9 ± 7.2	84.3 ± 6.6	0.06
PaO_2_, kPa	6.1 ± 0.9	6.4 ± 1.7	0.78
PaCO_2_, kPa	5.3 ± 1.3	5.4 ± 1.3	0.82
HR, bpm	119 ± 16	119 ± 18	0.84
Dyspnea, Borg	6.4 ± 1.5	6.5 ± 1.7	0.81
Leg fatigue, Borg	5.4 ± 2.0	5.2 ± 2.5	0.81
*Isotime*			
SpO_2_, %	82.8 ± 7.2	85.5 ± 6.7	0.01
PaCO_2_, kPa	5.3 ± 1.0	5.2 ± 1.1	0.63
HR, bpm	119 ± 16	118 ± 18	0.22

Data are presented as mean ± SD. CNC: Conventional Nasal Cannula; SpO_2_: arterial oxygen saturation by pulse oximetry; PaO_2_: arterial partial pressure of oxygen; PaCO_2_: arterial partial pressure of carbon dioxide; HR: heart rate; bpm: beats per minute; Borg: Score on the BorgCR10 scale

At end-exercise, patients rated a mean dyspnea score of 6.4 ± 1.5 and 6.5 ± 1.7 (p = 0.81) for the CNC and Oxymizer, respectively (Table 2). There were no significant differences between the two oxygen delivery systems for PCO_2_ and HR at isotime or at end-exercise.

## Discussion

In the current study, ILD patients with severe exercise-induced oxygen desaturation experienced a significant but modest increase in endurance time during CWRT when cycling with oxygen supplementation therapy provided through an oxygen conserving device (Oxymizer), compared with a conventional nasal cannula (CNC). Concerning the secondary outcomes (SpO_2_, PaCO_2_ and HR at isotime), there was a significantly higher SpO_2_ when cycling with the Oxymizer compared to the CNC, but there were no significant differences between the two oxygen supplementation sources for PaCO_2_ and HR.

**Fig 4:**
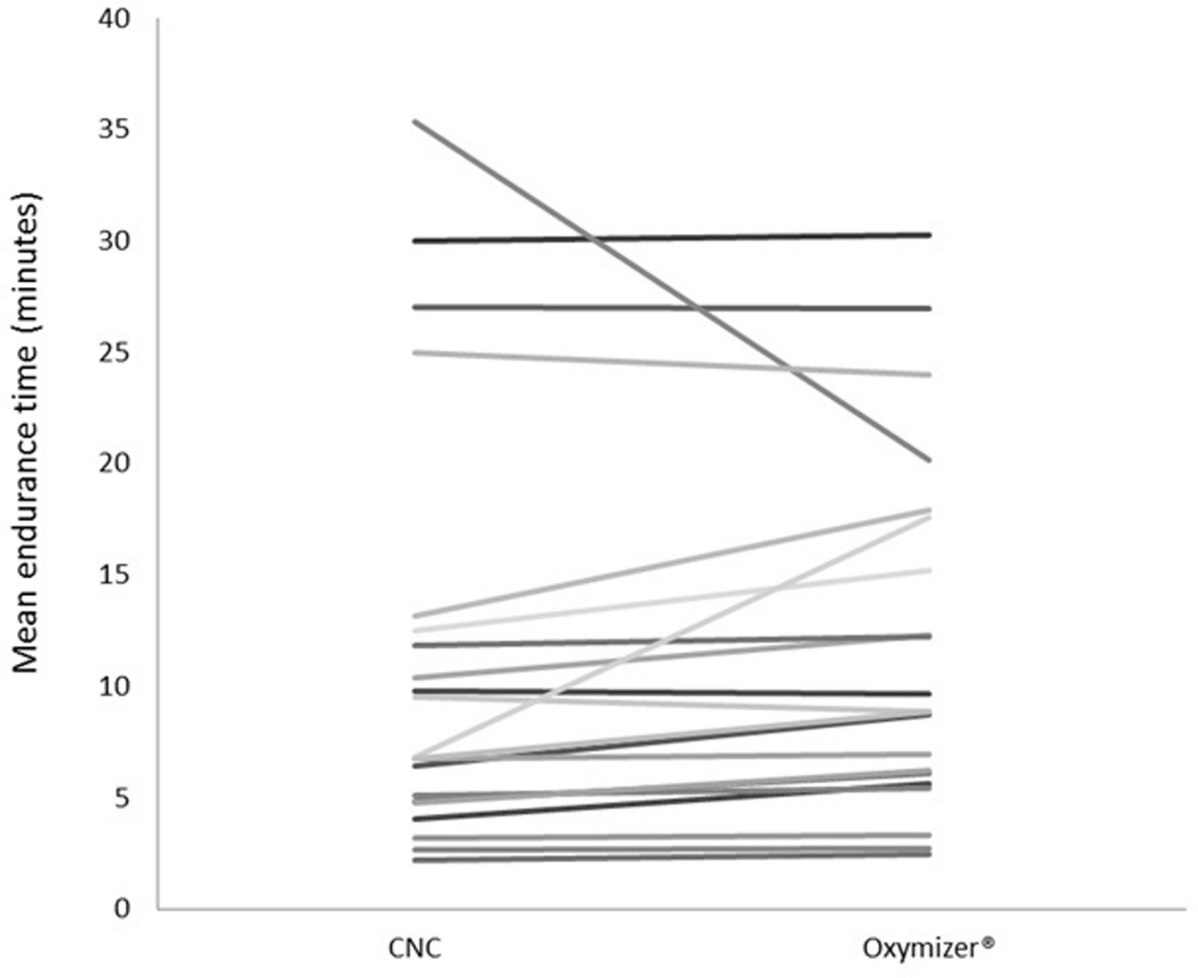
Individual mean endurance time for the two CWRT with CNC and Oxymizer respectively. CWRT: Constant work rate test; CNC: Conventional nasal cannula, n = 21.

A significant, but modest, mean increase of 38 seconds in cycle endurance time was found. Nevertheless, there was a large variation in response between CNC and Oxymizer ranging from –914 to 644 seconds (Fig 4). Indeed, 71% of the patients cycled longer using the Oxymizer, most probably due to higher SpO_2_. To date, it remains unclear why one-third of the ILD patients do not respond well to the use of the Oxymizer. The patients were instructed to breathe through their nose. However, the non-responders may still have breathed through their mouth, and therefore muddled the true effects of the Oxymizer. This means that healthcare professionals have to test which device is most effective and preferred in individual patients with ILD.

To our knowledge, there is no data on minimal important difference (MID) for cycle endurance time in patients with ILD [2]. Nevertheless, the ILD patients generally have severe exercise limitation, and even small increases in endurance capacity may represent a meaningful effect in the individual patient.

The patients reported equal levels of dyspnea at end-exercise for the Oxymizer. This may, due to the slightly longer endurance time, be interpreted as an advantage for the Oxymizer. In addition, there was a significant higher SpO_2_ at isotime and near significant higher SpO_2_ at end-exercise for the Oxymizer. ILD patients have a shallow and rapid breathing pattern. This might have underrepresented the effectiveness of the Oxymizer compared to the COPD patients [18].

There are a limited number of studies on oxygen supplementation therapy during exercise in patients with ILD. A recent Cochrane review summarize studies on oxygen supplementation compared to placebo [12]. Only three studies including a total of 98 patients with ILD were analyzed. In two of the studies, supplemental oxygen was not enough to prevent exercise desaturation, and there were no effects on exercise endurance or dyspnea [25].

In contrast, two retrospective observational studies on oxygen during exercise in 52 and 70 ILD patients respectively [26,27] suggests that oxygen supplementation therapy improves walking distance as well as end-exercise oxygen saturation and dyspnea. Mean SpO_2_ in these studies were below 88% and the oxygen flow rate was individually titrated to levels > 6 l/min. Therefore, further studies are needed to assess optimal criteria for prescribing oxygen during exercise in ILD patients and to assess whether response to different oxygen sources may differ according to type of ILD.

The individual oxygen flow given in the current study with both CNC and the Oxymizer during the CWRT ranged from 2 to 6 l/min. The flow rate was not sufficient to maintain SpO_2_ > 90%, which is the goal in oxygen therapy [7]. To our knowledge there is no evidence on the dose-response of supplemental oxygen during exercise in patients with ILD. The failure to achieve a SpO_2_ > 90% is in accordance with our study and is known from other studies on oxygen supplementation therapy during exercise in ILD [12,28]. This shows the difficulty to reach the oxygenation goal. One suggestion for further studies is in advance to titrate an individual exercise dose aimed to established SpO_2_ < 90% during the actual kind of activity.

The current study therefore wanted to evaluate if an older and sparsely used method could be an option for patients with ILD and severe exercise hypoxemia to achieve a SpO_2_ > 90%. There are few studies comparing CNC and Oxymizer on endurance time, SpO_2_ and dyspnea in patients with chronic lung disease. To our knowledge, there is just one study on patients with ILD [28] and two studies on COPD-patients [18,28]. Marti et al. [28] studied both patients with ILD and COPD, and found in 31 patients with ILD no differences in walking distance during a 6-minute walking test (6MWT) between the two oxygen sources, but the Oxymizer resulted in a significantly higher SpO_2_ at end-exercise compared to CNC. The results for the COPD patients are diverging. Gloeckl et al. studied 43 patients with COPD and found a significant higher cycling endurance time (858 vs. 766 s, p < 0.001) and a higher SpO_2_ at isotime using the Oxymizer (93.5 vs. 90.4%, p = 0.027) [18]. However, Marti et al. [28] found no further increase of 6MWT distance for the Oxymizer compared to the CNC for the 28 COPD-patients. This may be explained by the different exercise modes; cycling versus walking, where whole body exercise, as walking, may induce a more pronounced desaturation [29].

The current trial has some limitations. There was a wide range in endurance time and the intensity of 70% of peak work rate might have been to low. Four patients cycled for 25 minutes or more and may have terminated the task due to other reasons than exhaustion; e.g. getting bored. The current study was not designed to gather information about adherence or preferences on the use of Oxymizer compared to CNC. The Oxymizer cannula is somewhat thicker than a CNC, and may therefore be less preferred. In a study on patients on LTOT with a former and thicker model of Oxymizer, further use was declined in 43%, despite that they successfully maintained the SpO_2_ [30]. Further studies should also focus on the ease of use and the patient perspective.

## Conclusion

Generally, oxygen provided by an oxygen conserving nasal cannula (Oxymizer) during exercise significantly improved cycle endurance time and SpO_2_ at isotime in ILD patients compared to a conventional nasal cannula. However, the increase in cycle endurance time was modest and adequate oxygen saturation was not achieved for neither of the oxygen supplies and the Oxymizer did not improve dyspnea score compared to a CNC. Further studies are needed for evaluating oxygen delivery modalities during exercise in ILD patients with exercise-induced desaturation.

## Supporting information

S1 CONSORT checklist PlosOne. (PDF)

S1 Oxymizer-studie Ethikantrag. (PDF)

S1 Study protocol Oxymizer ILD study translated. (PDF)
