# Supplementary material for: A randomized cross-over trial on the direct effects of oxygen supplementation therapy using different devices on cycle endurance in hypoxemic patients with Interstitial Lung Disease
Source: PLoS One. 2018 Dec 28;13(12):e0209069. doi: 10.1371/journal.pone.0209069 (PMC6310247; doi:10.1371/journal.pone.0209069)
Supplement: S1 Oxymizer-studie — (PDF) [file pone.0209069.s002.pdf]

SCHÖN KLINIK
Berchtesgadener Land

Antrag an die Ethik-Kommission zur Beurteilung eines Forschungsvorhabens am Menschen

Titel:

„Effekte einer Sauerstoffsonde mit Reservoir (Oxymizer®) im Vergleich zu einer konventionellen Sauerstoffbrille bei Patienten mit COPD, Stadium III und IV oder fibrosierenden Lungenerkrankungen unter körperlicher Belastung“

Januar 2012

1. **Antragsteller**

Dr. med. Klaus Kenn

Schön Klinik Berchtesgadener Land

Fachzentrum für Pneumologie, Allergologie und Schlafmedizin

Malterhöh 1

83471 Schönau am Königssee

Tel.: 08652-93-1540

E-Mail: kkenn@schoen-kliniken.de

2. **Titel des Forschungsvorhabens**

„Effekte einer Sauerstoffsonde mit Reservoir (Oxymizer®) im Vergleich zu einer konventionellen Sauerstoffbrille bei Patienten mit COPD, Stadium III und IV oder fibrosierenden Lungenerkrankungen unter körperlicher Belastung“

3. **Ausbildungsdaten und Prüferfahrungen**

Ausbildungsdaten :

- 1982-1985 Chirurgische Ausbildung Universität Köln, II. Lehrstuhl und städtisches Krankenhaus Friedrichshafen
- 1985-1990 Innere Medizin, Krankenhaus Friedrichshafen
- 1990-1992 Innere Medizin, Pneumologie, Hochgebirgsklinik Davos, Schweiz
- 1992-1993 Pneumologie, Thoraxzentrum Löwenstein
- 1993-1997 Oberarzt Pneumologie, Hochgebirgsklinik Davos, Schweiz
- Seit 1997 Chefarzt Fachzentrum Pneumologie, Allergologie und Schlafmedizin in der Schön Klinik Berchtesgadener Land

Prüferfahrung:

- Verantwortliche Durchführung einer Phase-II-Studie mit Montelucast (intravenöse Anwendungen) bei Asthma bronchiale an der Hochgebirgsklinik Davos, Schweiz.
- Durchführung einer prospektiven Studie zur Prävalenz der Vocal Cord Dysfunction (VCD) als Differentialdiagnose zum schweren Asthma bronchiale, veröffentlicht beim Amerikanischen Pneumologenkongress 1997.
- Offene klinische Studien zur prä- und postoperativen Rehabilitation bei Lungentransplantationskandidaten.
- Klinische Studie zur Trainierbarkeit von Patienten mit interstitieller Lungenerkrankung: Vorstellung der Ergebnisse beim Europäischen Pneumologenkongress 2006.

- Teilnahme an einer Multicenter-Studie zur Erfassung der Effekte einer AHB im Bereich der Pneumologie, veröffentlicht: Deutsche Medizinische Wochenschrift 2006.
- Aktuell Teilnahme an einer klinischen Studie zur Evaluation von Stent-Implantationen, im Sinne einer endoskopischen Lungenvolumenreduktion (eLVR) in Kooperation mit der Klinik Hemer sowie dem Krankenhaus Bad Reichenhall.
- Teilnahme an klinischer Studie zur Erprobung von AV-Shuntanlage bei COPD-Patienten mit respiratorischer Insuffizienz
- Promotionsarbeit in Kooperation mit der Universität Marburg zum Thema: Angst und Depression bei fortgeschrittener COPD.
- Promotionsarbeit in Kooperation mit der Universität Marburg zum Thema: „End-of-life"-Ängste bei fortgeschrittenen Lungenerkrankungen.
- "Interval versus continuous training in patients with end stage lung disease before lung transplantation" (Glöckl, R; Weber-Lange, B; Halle, M; Kenn, K. ERS J 2009; 34: Suppl.53: 286)
- "Characteristics of oxygen responder before and after rehabilitation" (Heinzelmann, I; Kenn, K. AJRCCM 2010;181: A3580)
- Aktuelle Studie in Kooperation mit der Dt. Sporthochschule in Köln zum Thema: Effekte körperlichen Trainings auf die Quadrizeps-Genexpression bei COPD-Patienten im Vergleich zu Alpha-1-Antitrypsinmangel-Emphysem-Patienten.
- Veröffentlichung zu nicht-invasiver Beatmung: Köhnlein, T; Schönheit-Kenn, U; Winterkamp, S; Welte, T; Kenn, K. Non-invasive ventilation in pulmonary rehabilitation of COPD patients.
- Veröffentlichung zu Vocal cord dysfunction: Kenn, K; Balkissoon, R. Vocal cord dysfunction: What do we know? ERJ 2011; 37: 194-200.

4. Kooperationspartner

- **Zentrale Durchführung und Auswertung:**

  Schön Klinik Berchtesgadener Land
  Fachzentrum für Pneumologie, Allergologie und Schlafmedizin
  Malterhöh 1
  83471 Schönau am Königssee

5. Bei studienbedingten Strahlenbelastungen

- entfällt –

6. Wissenschaftliche Angaben zum Forschungsvorhaben

6.1 Fragestellung/ Studienziel

Einleitung

Bei Patienten mit fortgeschrittenen Lungenerkrankungen wie z.B. schwerstgradiger chronisch obstruktiver Lungenerkrankung (COPD Grad III und IV nach GOLD) oder fibrosierenden Lungenerkrankungen besteht häufig die Indikation zur Sauerstofflangzeittherapie (LTOT). Die Verordnung einer Sauerstofflangzeittherapie ist in nationalen und internationalen Leitlinien geregelt. Für Deutschland gelten die Empfehlungen der Deutschen Gesellschaft für Pneumologie (DGP).

Die Versorgung von Patienten mit LTOT ist apparativ auf verschiedene Art und Weise möglich. Neben der wenig praktikablen Versorgung mit Sauerstoff-(Stahl-)Flaschen sind Sauerstoffkonzentratoren und Flüssigsauerstoffgeräte im Einsatz. Im häuslichen Bereich kommen sowohl Konzentratoren wie auch Flüssigsauerstoffsysteme zur Anwendung, außer Haus vorwiegend letztere. Bei einigen der oben genannten schwerer erkrankten Patienten sind unter Belastung hohe Sauerstoffflussraten (>= 2L/min) notwendig, um eine ausreichende Oxygenierung zu erreichen. Dadurch wird die Mobilität (außer Haus) oftmals zeitlich sehr stark limitiert, da die vorhandenen tragbaren Geräte nur eine begrenzte Menge Flüssigsauerstoff beinhalten und dann wieder aufgefüllt werden müssen. Da keine flächendeckenden Nachfüllstationen existieren, müssen die Patienten dann ausreichend früh wieder in die Wohnung zurückkehren, um die tragbaren, mobilen Geräte erneut zu befüllen. Dies stellt über die Krankheitssymptome hinaus eine zusätzliche, krankheitsbedingte Einschränkung der Teilhabe am Alltagsleben und somit der Lebensqualität dar. Diese Einschränkung der Mobilität ist gerade bei höheren Flussraten von besonderer Bedeutung.

Eines der größten Probleme der oben beschriebenen Patienten ist eine krankheitsbedingte muskuläre Dekonditionierung, die die Mobilität ebenfalls stark einschränkt. Um diesem Kraftdefizit möglichst effizient entgegen zu wirken, wird postuliert, daß zusätzlich zu einer medizinischen Trainingstherapie im Rahmen der Rehabilitation eine ausreichende Oxygenierung sicher gestellt sein muss bzw. daß die Trainingseffekte um so besser sind, je besser die Oxygenierung ist.

Die Schön Klinik Berchtesgadener Land ist mit ca. 1200 stationär betreuten Patienten pro Jahr eine der führenden Kliniken in der Versorgung von Patienten mit fortgeschrittenen Lungenerkrankungen. Das multimodale Rehabilitationsprogramm beinhaltet neben der medizinischen Versorgung auch eine individualisierte Trainingstherapie, Atemtherapie, physikalische Maßnahmen, Ernährungsberatung sowie eine psychologische und

sozialmedizinische Betreuung. Die Überprüfung der Indikation für eine LTOT und die bedarfsadaptierte individuelle Einstellung für verschiedene Situationen (Ruhe, Schlaf und körperliche Belastung) ist neben der sich anschließenden Regelung der Kostenübernahme und der Einleitung einer apparativen Versorgung Standardbestandteil einer pneumologischen Rehabilitation.

Die Zufuhr des gasförmigen Sauerstoffs aus dem jeweiligen Gerät erfolgt im Regelfall über so genannte Sauerstoffsonden. Diese dünnen Kunststoffschläuche, die am Ende mit einer „Nasenbrille“ ausgestattet sind, gewährleisten die Einleitung des Sauerstoffs über die Nase in die Atemwege.
Der Oxymizer® ist eine spezielle, in den USA entwickelte Sauerstoffsonde, die neben einem größerlumigen Schlauch-/Nasenbrillensystem noch ein eingearbeitetes Reservoir enthält. Dadurch kann in der Einatmungsphase des Patienten eine deutlich höhere Sauerstoffkonzentration (FIO_2) appliziert werden. Der Oxymizer® stellt somit ein einfaches System zur Verbesserung der Oxygenierung dar.

Die Herstellung des Oxymizers® erfolgt durch die Firma CHAD-Therapeutics, Florida (siehe Anhang). Der Vertrieb in Deutschland erfolgt über die Firma Vivisol, Neufahrn. (Hilfsmittelnummer HMV 14.99.99.1031).

Das Gerät wird im Rahmen seiner Zweckbestimmung angewendet.

Stand der Forschung

Die positive Wirkung einer Sauerstofflangzeittherapie ist seit den frühen achtziger Jahren bekannt in den entsprechenden Leitlinien der nationalen und internationalen Fachgesellschaften geregelt und stellt somit eine pneumologisches Standardtherapieverfahren dar.
Der Oxymizer® wurde Mitte der 80er Jahre entwickelt und seine Anwendung in Ruhe, bei Belastung und in der Nacht als positiv für den Patienten beschrieben. Allerdings sind die damaligen Fallzahlen (n<=10) zu gering um heutigen wissenschaftlichen Ansprüchen zu genügen. Die in den nachfolgenden Jahren erschienene Literatur ist spärlich, so dass der Oxymizer® zumindest in Deutschland noch keinen hohen Bekanntheitsgrad erreicht hat.
In einer in unserem Haus in 2011 durchgeführten Pilot-Studie untersuchten wir 44 Patienten mit fibrosierender Lungenerkrankung und 20 COPD Patienten (Grad III und IV nach GOLD), die einen hohen Sauerstoffbedarf in Ruhe hatten, unter Ruhebedingungen.

Bei den Patienten mit fibrosierender Lungenerkrankung konnte bei gleichbleibendem Kohlendioxidpartialdruck ($paCO_2$) der kapilläre Sauerstoffpartialdruck (paO_2) unter Verwendung des Oxymizers® in Ruhe jeweils signifikant angehoben werden. Bei den COPD-Patienten zeigte sich ein positiver Trend ohne ein Signifikanz Niveau zu erreichen. Die vorläufigen Daten werden in diesem Jahr auf dem deutschen und amerikanischen Pneumologenkongress vorgestellt.

Art der Studie

Prospektiv, randomisiertes cross-over Design.

Forschungsvorhaben mit potentiellem Nutzen für den Teilnehmer

In der geplanten Studie soll an einer ausreichenden Anzahl von Patienten der Nutzen der Anwendung des Oxymizers® untersucht werden. In einem prospektiven, randomisierten cross-over-Design sollen alle eingeschlossenen Patienten mit fibrosierender Lungenerkrankung bzw. mit COPD Stadium III und IV nach GOLD in Ruhe und bei Belastung untersucht werden.
Die Auswirkungen auf die Belastbarkeitsdauer einerseits sowie das mögliche Einsparpotential bzgl. der verbrauchten Sauerstoffmenge und die damit abschätzbare Verlängerung der „Außer-Haus-Mobilität“ sollen untersucht werden.
Alle verwendeten Diagnostiken entsprechen regulären Verfahren der Rehabilitation in unserem Hause und stellen keinerlei zusätzliche Gefährdung dar.

Forschung an körpereigenen Materialien/ Gewebeentnahme für Studienzwecke

Um Veränderungen des paO_2, $paCO_2$ und des pH-Wertes im Blut vor,und nach der Ergometerbelastung sichtbar zu machen, erfolgt zu den genannten Zeitpunkten jeweils eine kapillare Blutgasanalyse. Zudem erfolgt mittels transcutaner Messung die kontinuierliche Erfassung von Sauerstoffsättigung (SaO_2) und des $paCO_2$ (Sentec-Messung).

Design der Studie

Studienplan

Alle teilnehmenden Probanden erhalten das gleiche multimodale Rehabilitationsprogramm inkl. medizinischer Trainingstherapie (MTT) wie es in unserer Klinik indikationsbezogen üblich ist.

Zur Bestimmung der körperlichen Leistungsfähigkeit und der Belastungstoleranz bei den unterschiedlichen Applikationsformen wird die maximale Belastungsdauer bei konstanter Intensität („endurance time“) (van´t Hul 2003) an den Tagen 7 und 14 gemessen.
Es erfolgt zuvor eine Randomisierung, die festlegt, ob am Tag 7 die erste Endurance time mittels konventioneller Sonde oder Oxymizer®-Sonde ermittelt wird. Am Tag 14 wechselt die Reihenfolge entsprechend. Die Untersuchungen werden in Anwesenheit eines Arztes unter fortlaufender Kontrolle von Blutdruck, EKG, SaO_2 und CO_2 (transcutan) durchgeführt.
Am Tag 7 werden im Rahmen der Ausgangswertbestimmung Blutgasanalysen in Ruhe zum Vergleich „normale Nasensonde“ versus Oxymizer® zur Findung einer „Äquivalenzflussrate“ durchgeführt. Hierbei erfolgt zunächst eine Messung der BGA mit konventioneller O_2-Sonde bei verordneter LTOT-Flussrate (=X). Daneben wird bei gleicher O_2-Flussrate eine BGA nach 10 Minuten Atmung über Oxymizer® durchgeführt. Anschließend wird der PaO_2 und $PaCO_2$ unter Oxymizer®-Anwendung mit Flussraten von X-1l und X-2l ermittelt. Es wird postuliert, dass unter Oxymizer® X- 1 oder X-2 eine vergleichbare Oxygenierung zu erzielen ist wie unter X bei konventioneller Sonde.
Für die sich anschließenden Belastungsuntersuchungen dient die am Tag 1 ermittelte Maximalleistung (W_{max}) als Grundlage zur Errechnung der Intensität für den Belastungsdauertest. Der Proband wird instruiert, im ersten Meßdurchgang so lange wie möglich bei 70% seiner maximalen Leistungsfähigkeit W_{max} auf dem Fahrradergometer bei mind. 50 U/Min. zu fahren. Die gemessene Zeit bis zum Abbruch der Belastung, die „endurance time“, dient als primärer Outcome-Parameter. Dieser Belastungstest ist valide und reagiert sensitiv auf kleine Veränderungen in der Belastbarkeit (LIT van´t Hul 2003). Bis zum zweiten Messdurchgang mit der jeweils anderen O_2-Sonde wird eine Erholungszeit von 2 Stunden berücksichtigt. (Siehe Protokollskizze)

Zur Charakterisierung des Kollektivs werden neben der Erfassung demographischer Daten Bodyplethysmographien und Spirometrien durchgeführt sowie zusätzlich die Diffusionskapazität (DLCO) gemessen.

Die Fragebögen geben ebenfalls prä und post Rehabilitation Aufschluss über die allgemeine und krankheitsspezifische Lebensqualität sowie über psychische Faktoren.

Studienablauf

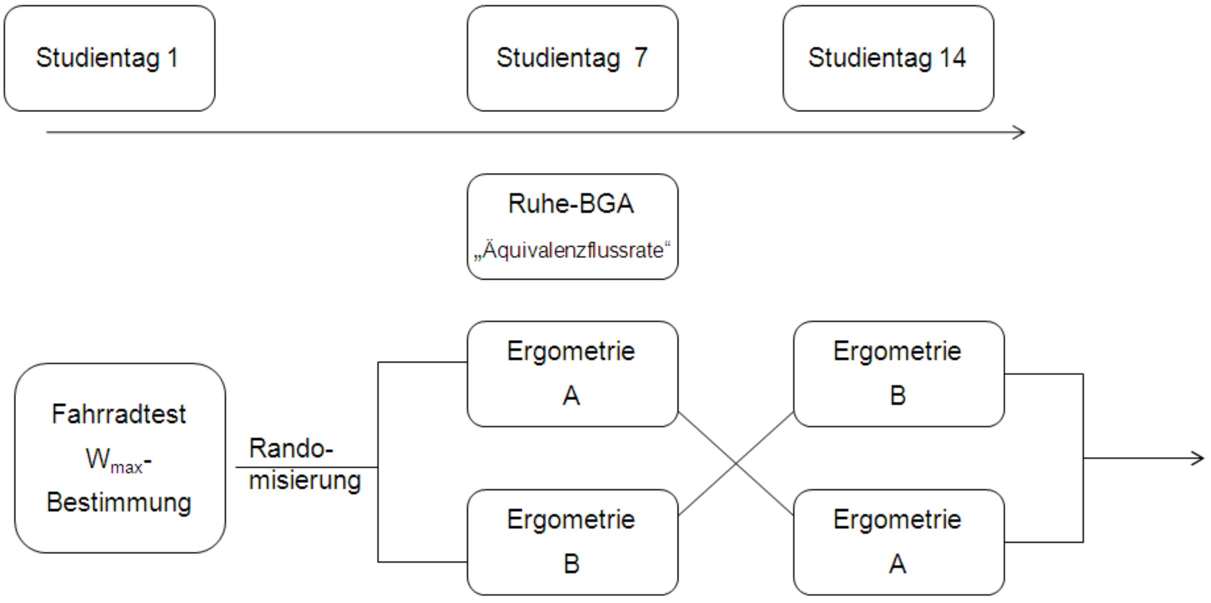

Ergometrie A oder B je nach Randomisierung

Einschlusskriterien:

- Stationärer Patient in der Schön Klinik Berchtesgadener Land
- COPD III oder IV oder fibrosierende Lungenerkrankung mit Indikation für eine LTOT und Flussraten >= 2L/min in Ruhe und/oder bei Belastung

Wegen des zu erwartenden geringen Benefits bezüglich der Ausdauerleistung durch eine Oxymizer®-Sonde unter Belastung für COPD-Patienten wird die Verteilung wie folgt gewählt: **COPD : Fibrose = 2 : 1**

Ausschlusskriterien:

- Akute kardiale Dekompensation, sowie die üblichen Kontraindikationen gegen eine Belastungsuntersuchung wie nicht ausreichend eingestellte arterielle Hypertonie, instabile Angina pectoris, höhergradige Herzklappenvitien, schwerer Lungenhochdruck, maligne Herzrhythmusstörungen.

Abbruchkriterien:

- Akuter Infekt oder andere gravierende akute Erkrankungen (gravierende Symptome länger als 3 Tage; Notwendigkeit der wesentlichen medikamentösen Therapieveränderung)
- Abbruchkriterien bei der Belastungsuntersuchung entsprechend medizinischen Standard (signifikante ST-Strecken-Senkungen oder –Hebungen, auftretende Angina-pectoris-Beschwerden, Blutdruck über 220 mmHg systolisch,

Blutdruckabfall unter den Ausgangswert oder höhergradigen ventrikulären Herzrhythmusstörungen.

- Verstöße gegen den Studienablauf, z.B. mangelnde Compliance

Wenn ein Patient aus der Studie ausscheidet, wird dies dokumentiert und führt zu keinen weiteren Folgen. Ein Steering- oder Safety Committee erscheint bei dieser Studienform nicht notwendig.

Hypothesen:

1. Die Endurance-time ist unter Verwendung des Oxymizers® größer als mit normaler Nasensonde.
2. Die Sauerstoffflussrate kann unter Verwendung des Oxymizers® in Ruhe und/oder bei Belastung reduziert werden, so dass die Außer-Haus-Mobilität des Patienten verbessert wird.

Biometrie:

- Die Fallzahlanalyse, errechnet anhand der Ergebnisse der o.g. Pilotstudie mit 50 COPD-Patienten und 25 Fibrosepatienten,.
- Mit Hilfe eines T-Tests für verbundene Stichproben wird die erreichte Dauer in der „endurance time“ (primärer Endpunkt) zwischen einer herkömmlichen Nasenbrille und dem Oxymizer® verglichen.
- Innerhalb einer Gruppe (COPD, Fibrose) werden die Sauerstoff- und Kohlendioxydpartialdrucke unter Verwendung der beiden Sauerstoffsonden und der unterschiedlichen Flussraten verglichen

**Untersuchungen/ Fragebögen**	**Tag1**	**Tag7**	**Tag 14**	**Entlassung**
Fahrrad-Stufentest zur Bestimmung der Endurance-time<br>mit Flussrate = Ruheflussrate + 2L/min	X			
2x endurance time (Ergometrie) bei 70% der Maximalleistung<br>nach Randomisation<br>Flussrate = Ruheflussrate + 2L/min		X*	x	
Fragebogen SF 36 (allg. Lebensqualität), HADS-Fragebogen	X			X
Fragebogen CRQ (organspezifische Lebensqualität)	X			X
Diffusionskapazität (DLCO)	X			
Bodyplethysmographie, Blutgasanalyse	X			X
Laborparameter (Routinelabor)	X			X
Bioimpedanzanalyse (BIA)	X			X

*gleiche Tageszeit ± 1 Std.

Beginn und Dauer der Studie:

- März 2012, Dauer ca. 1,5 Jahre

7. Diskussion ethisch-rechtlich relevanter Probleme

In der geplanten Studie findet keine Verblindung, Placebo-Verwendung oder das Vorenthalten einer Maßnahme statt. Es ist sowohl für Teilnehmer der Kontroll- als auch der Interventionsgruppe ein positiver Effekt auf die allgemeine Lebensqualität und die Leistungsfähigkeit zu erwarten.

Da alle Teilnehmer in der Schön Klinik Berchtesgadener Land zur stationären Rehabilitation untergebracht sind, ist im unwahrscheinlichen Fall des Auftretens von Nebenwirkungen jederzeit ein medizinischer Bereitschaftsdienst vor Ort.

Es werden sowohl mündliche als auch schriftliche Aufklärungen durchgeführt und eine schriftliche Einverständniserklärung eingeholt. Das Einverständnis kann der Teilnehmer jederzeit ohne Angabe von Gründen mündlich widerrufen.

7.1 Nicht-Einwilligungsfähigkeit

Bei fehlender Aufklärungs- oder Einwilligungsfähigkeit kommt keine Studienteilnahme in Frage.

7.2 Forschung an Minderjährigen

-entfällt-

7.3 Datenschutz

Zur Wahrung des Datenschutzes werden sämtliche Daten durch den Studienleiter bzw. durch beauftragte Mitarbeiter dokumentiert und pseudonymisiert. Die gesetzlichen Vorgaben des Datenschutzes werden im Rahmen dieser Studie selbstverständlich eingehalten. Die Ausführungen zum Datenschutz sind in der Patientenaufklärung enthalten, zusätzlich wird dem Patienten eine gesonderte datenschutzrechtliche Einverständniserklärung ausgehändigt.

8. Versicherung

Eine zusätzliche Versicherung erscheint nicht notwendig.

9. Finanzierung

Die geplante Studie wird lediglich durch kostenlose Bereitstellung von 75 Oxymizern® durch die Fa. Vivisol unterstützt.

Es ist geplant die Studie in Kooperation mit der Philipps-Universität Marburg durchzuführen.

Abschließend sei darauf hingewiesen, dass die Einholung eines Ethik-Kommission-Votums v.a. deshalb erfolgt, weil die Daten publiziert werden sollen und dieses Vorgehen internationalem Standard entspricht.

Für weitere Auskünfte steht der Unterzeichner jederzeit gerne zur Verfügung.

Dr. med. Klaus Kenn
Chefarzt Pneumologie
Klinikum Berchtesgadener Land

Literatur:

1. Domingo E. Evaluation of the use of three different devices for nocturnal oxygen therapy in COPD patients. Respiration. 1996;63(4):230-5.
2. Hagarty EM, Skorodin MS, Stiers WM, Mamdani MB, Jessen JA, Belingon EC. Performance of a reservoir nasal cannula (Oxymizer®) during sleep in hypoxemic patients with COPD. Chest. 1993 Apr;103(4):1129-34.
3. Vilsvik JS, Dahl B, Sailer R. [Oxygen conserving nasal catheters. Oxymizer® pendant]. Tidsskr Nor Laegeforen. 1992 Nov 30;112(29):3659-62..
4. Stewart AG, Howard P. Devices for low flow O2 administration. Eur Respir J. 1990 Jul;3(7):812-7.
5. Haber H, Raber W, Kapfhammer G, Vetter N. [Comparison of an oxygen-conserving module "Oxytron" and the reservoir cannula "Oxymizer® Pendant" with continuous oxygen administration via nasal prong in hypoxemic patients]. Wien Klin Wochenschr. 1990 May 25;102(11):325-9..
6. Collard P, Wautelet F, Delwiche JP, Prignot J, Dubois P. Improvement of oxygen delivery in severe hypoxaemia by a reservoir cannula. Eur Respir J. 1989 Sep;2(8):778-81.
7. Tiep BL, Burns M, Hererra J. A new pendant oxygen-conserving cannula which allows pursed lips breathing. Chest. 1989 Apr;95(4):857-60.
8. Evans TW, Waterhouse JC, Suggett AJ, Howard P. A conservation device for oxygen therapy in COPD. Eur Respir J. 1988 Dec;1(10):959-61.
9. Arlati S, Rolo J, Micallef E, Sacerdoti C, Brambilla I. A reservoir nasal cannula improves protection given by oxygen during muscular exercise in COPD. Chest. 1988 Jun;93(6):1165-9.
10. Gould GA, Hayhurst MD, Scott W, Flenley DC. Clinical assessment of oxygen conserving devices in chronic bronchitis and emphysema. Thorax. 1985 Nov;40(11):820-4.
11. Moore-Gillon JC, George RJ, Geddes DM. An oxygen conserving nasal cannula. Thorax. 1985 Nov;40(11):817-9.

Anhang:

Dem Oxymizer® beiliegende Patienteninformation:

OXYMIZER® PENDANT

DISPOSABLE OXYGEN-CONSERVING DEVICES

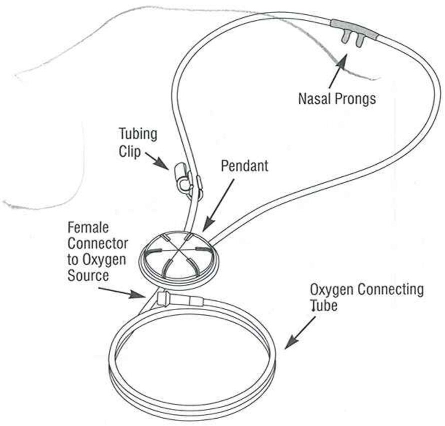

USER DIRECTIONS:

Your physician has prescribed the OXYMIZER Pendant oxygen-conserving device for you. Used properly, this device will provide you with the oxygen you need, as well as these advantages:

- It will take a lower flow rate to get the same amount of oxygen into your body - as much as 75% less, depending on the flow rate, than a standard nasal cannula.
- If you use a portable oxygen system, it will last longer, so you can be away from your main oxygen source longer. Or, you may be able to use a smaller, lighter weight portable system.

It is important that you follow your physician's instructions carefully concerning:

- How often and how long you receive oxygen from the system.
- The flow rate to use. (NOTE: the flow rate indicated for use with the OXYMIZER Pendant device may be 1/4-1/2 that prescribed with a non-oxygen-conserving cannula).
- When to replace your OXYMIZER Pendant device.

HOW TO PUT ON THE OXYMIZER PENDANT

1. Uncoil the tubing completely. Hold the device so that the front of the Pendant is facing away from you. *(Note: The front of the Pendant is designated with slots.)*

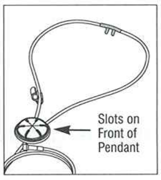

2. Position the nasal prongs so that they fit comfortably into your nostrils. The prongs should extend well into and pointing toward the back of your nose.

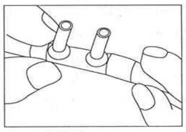

3. Position the nasal prongs and extend the tubing across your cheekbones.

4. Place the tubing around your ears and adjust each side so that you have a snug, comfortable fit.

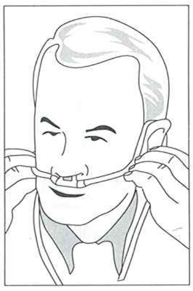

5. Attach tubing clip to join both sides of cannula tubing. Slide the clip upward beneath your chin to hold the tubing in place. *(You may choose not to use the clip by simply removing it.)*

6. The Pendant portion should rest against your chest below your throat. You may want to tuck it under your shirt or blouse.

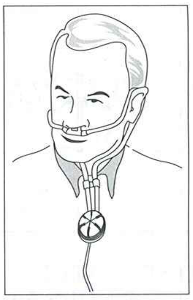

7. Connect the female connector to your oxygen source.

8. Turn on the oxygen and set the flow rate as prescribed by your doctor or health care professional. (NOTE: The flow rate prescribed for use with this device may be 1/4-1/2 that prescribed with a non-oxygen-conserving cannula.)

REPLACEMENT:

The OXYMIZER Pendant device should be replaced after approximately three weeks. More frequent replacement may be needed for cleanliness and sanitation, depending on conditions of use.

TODAY'S DATE:	REPLACE ON:

HUMIDIFICATION UNNECESSARY

Since less total oxygen is passing over the nasal mucosa, the drying effect of oxygen is significantly less and supplementary humidification can usually be eliminated. Since excessive moisture could impede the membrane action of the OXYMIZER Pendant device, use with humidifiers should be avoided.

You may also be interested in the original OXYMIZER oxygen-conserving device with the oxygen-saving mechanism contained in a facepiece, which is ideal for use at home.

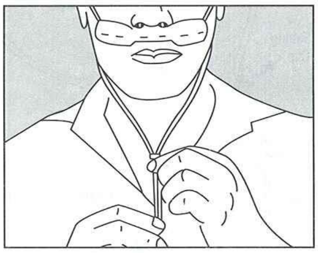

OXYMIZER® PENDANT

DISPOSABLE OXYGEN-CONSERVING DEVICES

CODE #P-224
FOR USE IN OXYGEN THERAPY

DISPOSABLE -
FOR SINGLE PATIENT USE ONLY
DIRECTIONS FOR USE ENCLOSED

CONTENTS: One (1) OXYMIZER Pendant disposable oxygen-conserving device with curved tubular nasal prongs, six (6) feet of connecting tube, and a female connector.

Manufactured for:
CHAD® THERAPEUTICS
A Division of Inovo, Inc.
2975 Horseshoe Drive South, Suite 600
Naples, FL 34104
1.800.423.8870

CE

2010

Lot#: 105095

Rx Only

U.S.Pat. Pending.
Made in China.
©2008 CHAD Therapeutics.

P/N AL-00024/08/C
