# Supplementary material for: A randomized cross-over trial on the direct effects of oxygen supplementation therapy using different devices on cycle endurance in hypoxemic patients with Interstitial Lung Disease
Source: PLoS One. 2018 Dec 28;13(12):e0209069. doi: 10.1371/journal.pone.0209069 (PMC6310247; doi:10.1371/journal.pone.0209069)
Supplement: S1 Study protocol — (PDF) [file pone.0209069.s003.pdf]

Application to the Ethics Committee for the assessment of a research project in humans

"Effects of an Oxygen Probe with a Reservoir (Oxymizer®) Compared to Conventional Oxygen Glasses in Patients with COPD, Stage III and IV, or Pulmonary Pulmonary Disease Under Physical Exposure"

January 2012

1. Applicant

Dr. med. Klaus Kenn
Schön Klinik Berchtesgadener Land
Center for Pulmonology, Allergology and Sleep Medicine
Malterhöh 1
83471 Schönau am Königssee
Tel: 08652-93-1540
E-Mail: kkenn@schoen-kliniken.de

2. Title of the research project

"Effects of an Oxygen Probe with a Reservoir (Oxymizer®) Compared to Conventional Oxygen Glasses in Patients with COPD, Stage III and IV, or Pulmonary Disease Under Physical Exposure"

3. Employment and study experience

Employment:

- 1982-1985 Surgical Education University of Cologne, II. Chair and City Hospital Friedrichshafen
- 1985-1990 Internal Medicine, Hospital Friedrichshafen
- 1990-1992 Internal Medicine, Pulmonology, Mountain Clinic Davos, Switzerland
- 1992-1993 Pulmonology, Thoraxzentrum Löwenstein
- 1993-1997 senior physician pulmonology, high mountain clinic Davos, Switzerland
- Since 1997 Senior Consultant Center for Pneumology, Allergology and Sleep Medicine at the Schön Klinik Berchtesgadener Land

Study experience:

- Responsible for conducting a Phase II study with montelucast (intravenous applications) in bronchial asthma at Davos Mountain Hospital, Switzerland.
- Conducted a prospective study on the prevalence of vocal cord dysfunction (VCD) as a differential diagnosis of severe bronchial asthma, published at the 1997 American Pulmonary Congress.
- Open clinical trials for pre- and postoperative rehabilitation of lung transplant candidates.
- Clinical study on the trainability of patients with interstitial lung disease: Presentation of the results at the European Congress of Pneumonia 2006.
- Participation in a multicenter study to assess the effects of AHB in the field of pneumology, published: Deutsche Medizinische Wochenschrift 2006.
- Currently participating in a clinical study evaluating stent implantation in terms of endoscopic lung volume reduction (eLVR) in cooperation with the Hemer Hospital and the Bad Reichenhall Hospital.
- Participation in a clinical trial to test AV shunt placement in COPD patients with respiratory failure
- Doctoral thesis in cooperation with the University of Marburg on the subject: anxiety and depression in advanced COPD.
- Doctoral thesis in cooperation with the University of Marburg on the topic: "End-of-life" anxiety in advanced lung diseases.
- "Interval versus continuous training in patients with end-stage lung disease transplantation" (Glöckl, R; Weber-Lange, B; Halle, M; Kenn, K. ERS J 2009; 34: Suppl.53: 286)

• "Characteristics of oxygen responders before and after rehabilitation" (Heinzelmann, I; Kenn, K. AJRCCM 2010; 181: A3580)
• Current study in cooperation with Dt. Sports University in Cologne on the topic: Effects of physical training on quadriceps gene expression in COPD patients compared to alpha-1-antitrypsin deficient emphysema patients.
• Publication on non-invasive ventilation: Köhnlein, T; Beauty Kenn, U; Winter Kamp, S; Welte, T; Kenn, K. Non-invasive ventilation in pulmonary rehabilitation of COPD patients.
• Publication on vocal cord dysfunction: Kenn, K; Balkissoon, R. Vocal cord dysfunction: What do we know? ERJ 2011; 37: 194-200.

4. Cooperation Partner
Central implementation and evaluation:
Schön Klinik Berchtesgadener Land
Center for Pulmonology, Allergology and Sleep Medicine
Malterhöh 1
83471 Schönau am Königssee

5. Study related radiation exposure
Not applicable.

6. Scientific information on the research project
6.1 Research question / study objective
Introduction
In patients with advanced lung diseases, e.g. severe chronic obstructive pulmonary disease (COPD grade III and IV after GOLD) or fibrotic lung disease is often the indication for long-term oxygen therapy (LTOT). The regulation of an oxygen long-term therapy is regulated in national and international guidelines. For Germany, the recommendations of the German Society of Pneumology (DGP) is applied.
The care of patients with LTOT is possible in various ways. In addition to the less practical supply of oxygen (steel) bottles, oxygen concentrators and liquid oxygen devices are in use. At home, both concentrators and liquid oxygen systems are used, with the exception of the latter mainly the latter. For some of the more severely ill patients mentioned above, high oxygen flow rates (> = 2L / min) are required under exercise to achieve adequate oxygenation. As a result, the mobility (outside the home) is often very limited in time, since the existing portable devices contain only a limited amount of liquid oxygen and then have to be replenished. Since there are no nationwide refill stations, patients need to return to the home sufficiently early to replenish the portable, mobile devices. In addition to the disease symptoms, this represents an additional, disease-related restriction of participation in everyday life and thus the quality of life. This restriction of mobility is of particular importance especially at higher flow rates.
One of the biggest problems of the patients described above is a disease-related muscular deconditioning that also severely limits mobility. To counteract this power deficit as efficiently as possible, it is postulated that in addition to a medical training therapy in the context of rehabilitation sufficient oxygenation must be ensured.

The Schön Klinik Berchtesgadener Land is one of the leading clinics in the care of patients with advanced lung diseases with approximately 1,200 inpatients per year. In addition to medical care, the multimodal rehabilitation program also includes individualized training

therapy, respiratory therapy, physical measures, nutritional counseling and psychological and socio-medical care. The indication for LTOT and the individual adjustment for different situations (rest, sleep and physical exertion) is a standard component of a pneumological rehabilitation in addition to the subsequent regulation of reimbursement of costs and the initiation of care.

The supply of gaseous oxygen from the respective device is usually via so-called oxygen probes. These thin plastic tubes, which end with a "nasal cannula", ensure the delivery of oxygen through the nose into the airways.
The Oxymizer® is a special, developed in the US, oxygen probe, which in addition to a larger-lumen hose / nasal cannula system also contains an incorporated reservoir. As a result, a significantly higher oxygen concentration (FIO2) can be applied in the patient's inhalation phase. The Oxymizer® is thus a simple system for improving oxygenation.

The Oxymizers® are manufactured by CHAD-Therapeutics, Florida (see Appendix). Sales in Germany are made by Vivisol, Neufahrn. (Aid number HMV 14.99.99.1031).
The device is used in its intended purpose.

State of research

The positive effect of long-term oxygen therapy has been well-known since the early eighties (in the guidelines of the national and international medical societies) and thus represents a standard pneumological therapy.
The Oxymizer® was developed in the mid-1980s and described its use as positive for the patient at rest, during exercise and at night. However, the case numbers at that time (n <= 10) are too low to meet today's scientific demands. The literature published in the following years is sparse, so that the Oxymizer®, at least in Germany, has not achieved a high degree of popularity.
In a pilot study conducted in our facility in 2011, we studied 44 patients with fibrotic lung disease and 20 COPD patients (grade III and IV GOLD) who had a high oxygen demand at rest under resting conditions. In the patients with fibrosing lung disease, the capillary oxygen partial pressure (paO2) using the Oxymizers® at rest could be increased significantly at rest while maintaining the carbon dioxide partial pressure (paCO2). COPD patients showed a positive trend without reaching a significance level. The preliminary data will be presented this year at the German and American Pulmonology Congress.

Type of study

Prospective, randomized crossover design.

Research projects with potential benefits for the participant

The planned study will evaluate the benefits of using the Oxymizers® on a sufficient number of patients. In a prospective, randomized cross-over design, all patients included with fibrosing lung disease or with COPD stage III and IV after GOLD should be examined at rest and during exercise.
The effects on the duration of exposure on the one hand, and the possible savings potential with regard to the amount of oxygen consumed and the estimated extension of "out-of-home mobility" should be investigated.

All used diagnostics correspond to regular procedures of the rehabilitation in our house and represent no additional danger.

Research on endogenous materials / tissue sampling for study purposes
In order to visualize changes in the paO2, paCO2 and the pH value in the blood before and after the ergometer load, a capillary blood gas analysis is carried out at each time. In addition, transcutaneous measurement is used to continuously record oxygen saturation (SaO2) and paCO2 (sentec measurement).

Design of the study
Study plan
All participating subjects receive the same multimodal rehabilitation program including medical training therapy (MTT) as is usual in our clinic according to the indication.

To determine the physical performance and the exercise tolerance in the different forms of application, the maximum duration of endurance ("endurance time") (van't Hul 2003) on days 7 and 14 is measured.
A randomization is performed to determine whether the first endurance time is determined on day 7 using a conventional probe or Oxymizer® probe. On day 14, the order changes accordingly. The examinations are performed in the presence of a physician under continuous control of blood pressure, ECG, SaO2 and CO2 (transcutaneous).
On day 7, baseline blood gas analyzes are performed to compare "normal nasogastric tube" vs. Oxymizer® to find an "equivalent flow rate". First, the BGA is measured with a conventional O2 probe at a prescribed LTOT flow rate (= X). In addition, with the same O2 flow rate, a BGA is carried out after 10 minutes of breathing via Oxymizer®. Subsequently, the PaO2 and PaCO2 are determined under Oxymizer® application with flow rates of X-1l and X-2l. It is postulated that under Oxymizer® X-1 or X-2 a comparable oxygenation can be achieved as under X in conventional probe.
For the subsequent exercise tests, the maximum power (Wmax) determined on day 1 serves as the basis for calculating the intensity for the exercise duration test. The subject is instructed, in the first test period as long as possible at 70% of its maximum performance Wmax on the bicycle ergometer at least 50 U / min. to drive. The measured time until termination of the exercise, the "endurance time", serves as a primary outcome parameter. This exercise test is valid and sensitive to small changes in exercise capacity (LIT van't Hul 2003). A recovery time of 2 hours is taken into account until the second measurement run with the other O2 probe. (See protocol sketch)

To characterize the patients, bodyplethysmography and spirometry are performed in addition to the collection of demographic data. In addition the diffusion capacity (DLCO) is measured.

The questionnaires will give pre and post rehabilitation information about the general and disease-specific quality of life as well as about mental factors.

Studienablauf

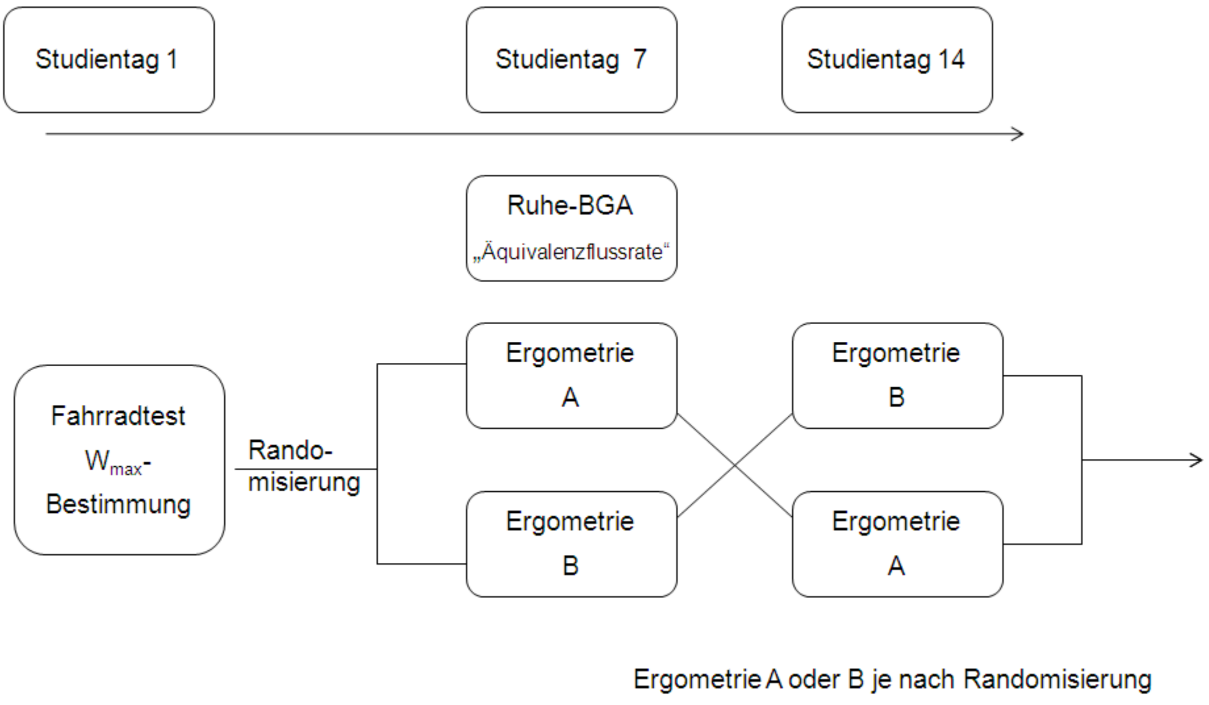

Inclusion criteria

• Inpatient at the Schön Klinik Berchtesgadener Land
• COPD III or IV or fibrotic lung disease with indication of LTOT and flow rates> = 2L / min at rest and / or during exercise. Because of the expected low benefit in terms of endurance performance by an Oxymizer® probe under stress for COPD patients, the distribution is chosen as follows: COPD: fibrosis = 2: 1

Exclusion criteria

• Acute cardiac decompensation, as well as the usual contraindications to exercise examinations such as inadequate arterial hypertension, unstable angina pectoris, high-grade heart valve defects, severe pulmonary hypertension, malignant cardiac arrhythmias.
Termination criteria:
• Acute infection or other serious acute illnesses (serious symptoms lasting more than 3 days, necessity of essential medical therapy change)
• Medical Exposure Demand Termination criteria (significant ST segment depression or exertion, angina pectoris complaints, systolic blood pressure above 220 mmHg, baseline blood pressure drop, or higher ventricular arrhythmia).
• Violations of the course of studies, e.g. lack of compliance. If a patient leaves the study, this will be documented and will result in no further consequences. A steering or safety committee does not seem necessary in this study.

Hypotheses

1. Endurance-time is greater when using the Oxymizer® than with a normal nasogastric tube.
2. The oxygen flow rate may be reduced at rest and / or during exercise using the Oxymizers® to improve the patient's mobility away from home.

Biometrics

• The sample size analysis, calculated on the basis of the results of the o.g. Pilot study with 50 COPD patients and 25 fibrosis patients.

• Using a connected sample T-test, compare the duration achieved in the "endurance time" between a conventional nasal cannula and the Oxymizer®.

• Within one group (COPD, fibrosis), the oxygen and carbon dioxide partial pressures are compared using the two oxygen probes and the different flow rates.

Examinations/Questionnaires	Day1	Day7	Day14	At discharge
Incremental cycle test to determine the peak work rate (PWR)	X			
2x cycling endurance test at 70% of PWR using either CNC or Oxymizer depending on randomization		X*	X	
Questionnaire SF 36 (general quality of life), HADS-Questionnaire	X			X
Questionnaire CRQ (organ-specific quality of life)	X			X
DLCO	X			
Bodyplethysmography, blood gas analysis	X			X
Standard laboratory parameters	X			X
Biological impedance analysis (BIA)	X			X

*same time of the day ± 1 hour.

Start and duration of the study

• March 2012, duration approx. 1.5 years

7. Discussion of ethically-legally relevant problems

The study have no blinding or placebo use. Both participants in the control and intervention groups are expected to have a positive effect on overall quality of life and performance. Since all participants are accommodated in the Schön Klinik Berchtesgadener Land for inpatient rehabilitation, in the unlikely event of the occurrence of side effects is always a medical on-call service on site.
Both verbal and written explanations are carried out and a written consent is obtained. The consent can be revoked verbally by the participant at any time without giving reasons.

7.1 Non-consent

If there is no informed consent, study participation is not an option.

7.2 Research on minors

Not applicable

7.3 Privacy

To protect data protection, all data are documented and pseudonymized by the study director or by authorized staff. The legal requirements of data protection are, of course, complied with in the context of this study. The statements on data protection are included in the patient information, in addition, the patient is given a separate data protection consent form.

8. Insurance
An additional insurance does not seem necessary.

9. Financing
The planned study is only supported by the provision of 75 Oxymizern® by Vivisol.

It is planned to carry out the study in cooperation with the Philipps-University Marburg.

Finally, it should be noted that obtaining an ethics committee vote is necessary. This is because the data is to be published and this procedure conforms to international standards.

For further information the signer is always at your disposal.

Dr. med. Klaus Kenn
Chief physician pneumology
Hospital Berchtesgadener Land

Literature:

1. Domingo E. Evaluation of the use of three different devices for nocturnal oxygen therapy in COPD patients. Respiration. 1996;63(4):230-5.
2. Hagarty EM, Skorodin MS, Stiers WM, Mamdani MB, Jessen JA, Belingon EC. Performance of a reservoir nasal cannula (Oxymizer®) during sleep in hypoxemic patients with COPD. Chest. 1993 Apr;103(4):1129-34.
3. Vilsvik JS, Dahl B, Sailer R. [Oxygen conserving nasal catheters. Oxymizer® pendant]. Tidsskr Nor Laegeforen. 1992 Nov 30;112(29):3659-62..
4. Stewart AG, Howard P. Devices for low flow O2 administration. Eur Respir J. 1990 Jul;3(7):812-7.
5. Haber H, Raber W, Kapfhammer G, Vetter N. [Comparison of an oxygen-conserving module "Oxytron" and the reservoir cannula "Oxymizer® Pendant" with continuous oxygen administration via nasal prong in hypoxemic patients]. Wien Klin Wochenschr. 1990 May 25;102(11):325-9..
6. Collard P, Wautelet F, Delwiche JP, Prignot J, Dubois P. Improvement of oxygen delivery in severe hypoxaemia by a reservoir cannula. Eur Respir J. 1989 Sep;2(8):778-81.
7. Tiep BL, Burns M, Hererra J. A new pendant oxygen-conserving cannula which allows pursed lips breathing. Chest. 1989 Apr;95(4):857-60.
8. Evans TW, Waterhouse JC, Suggett AJ, Howard P. A conservation device for oxygen therapy in COPD. Eur Respir J. 1988 Dec;1(10):959-61.
9. Arlati S, Rolo J, Micallef E, Sacerdoti C, Brambilla I. A reservoir nasal cannula improves protection given by oxygen during muscular exercise in COPD. Chest. 1988 Jun;93(6):1165-9.
10. Gould GA, Hayhurst MD, Scott W, Flenley DC. Clinical assessment of oxygen conserving devices in chronic bronchitis and emphysema. Thorax. 1985 Nov;40(11):820-4.
11. Moore-Gillon JC, George RJ, Geddes DM. An oxygen conserving nasal cannula. Thorax. 1985 Nov;40(11):817-9.
